# The diamidine DB75 targets the nucleus of *Plasmodium falciparum*

**DOI:** 10.1186/1475-2875-8-104

**Published:** 2009-05-14

**Authors:** Anne E Purfield, Richard R Tidwell, Steven R Meshnick

**Affiliations:** 1 Department of Microbiology and Immunology, School of Medicine, University of North Carolina, Chapel Hill, North Carolina, USA; 2 Department of Pathology and Laboratory Medicine, School of Medicine, University of North Carolina, Chapel Hill, North Carolina, USA

## Abstract

**Background:**

DB289, [2,5-bis(4-amidinophenyl)furan *bis-O*-methylamidoxime], is a broad spectrum anti-parasitic compound which has been shown to be effective against malaria in recent clinical trials. DB75, [2,5-bis(4-amidinophenyl)furan], is the active metabolite of this drug. The objective of this study was to determine the mechanism of action of DB75 in *Plasmodium falciparum*.

**Methods:**

Live parasites were observed by confocal microscopy after treatment with organelle specific dyes and DB75, an inherently fluorescent compound. Parasites were exposed to DB75 and assessed for growth and morphological changes over time using blood smears and light microscopy. Also, to determine if DB75 affects gene transcription, real time PCR was used to monitor transcript levels over time for six developmentally expressed genes, including *trophozoite antigen R45-like *(PFD1175w), *lactate dehydrogenase *(PF13_0141), *DNA primase *(PFI0530c), *isocitrate dehydrogenase *(PF13_0242), *merozoite surface protein-1 *(PFI1475w), and *merozoite surface protein-7 *(PF13_0197).

**Results:**

The results show that DB75 localizes in the parasite nucleus but not in other organelles. Once rings are exposed, parasites mature to the trophozoite stage and stall. No stage-dependent or gene-specific inhibition of transcription was seen. However, DB75 delayed peak transcription of trophozoite-stage genes.

**Conclusion:**

Taken together, DB75 appears to concentrate in the nucleus and delay parasite maturation.

## Background

Resistance to classical anti-malarials exists throughout the developing world [1]. Most of the current anti-malarials belong to a few classes of compounds for which cross-resistance exists. New anti-malarials with unique targets are needed to overcome cross-resistance.

Pentamidine, [1,5-di(4-amidinophenoxy)pentane], a dicationic diamidine, has been used for clinical treatment of protozoa infections such as leishmaniasis and Human African Trypanosomiasis (HAT) [2]. It also has activity against *Plasmodium falciparum*, but pentamidine is not orally bioavailable and has considerable side effects, including renal toxicity and cardiotoxicity [3-5]. Therefore, it has been passed over as a potential malaria therapeutic in favour of drugs with oral bioavailability and fewer side effects [5]. New pentamidine structural analogues have been synthesized and represent a class of broad-spectrum antimicrobials lacking the side effects associated with pentamidine [6,7]. The aromatic diamidines inspired by pentamidine are being investigated for therapeutic use with HAT, malaria, *Pneumocystis jiroveci *pneumonia (PCP) and leishmaniasis [2,6].

The dicationic diamidine, DB75, [2,5-bis(4-amidinophenyl)furan], is a fluorescent structural analogue of pentamidine with antimicrobial activity, but with little oral bioavailability [6,8,9]. Pafuramidine, also known as DB289, [2,5-bis(4-amidinophenyl)furan *bis-O*-methylamidoxime], is an orally active pro-drug of DB75 [7,10,11]. In a clinical trial of Thai patients with falciparum malaria, DB289 monotherapy (100 mg, twice daily for 5 days) exhibited a 96% (22 of 23) cure rate [10].

The target and mechanism of action of diamidines against malaria are currently unknown, but several possible mechanisms have been proposed. First, Stead *et al *showed that pentamidine is found in the food vacuole of *P. falciparum *where it binds toxic haem and inhibits formation of non-toxic haemozoin [12]. Second, DNA-containing organelles are also potential targets. In *Trypanosoma brucei gambiense*, DB75 localizes in DNA-containing organelles, such as the nucleus and kinetoplast [13,14]. Further, nucleic acid-binding is believed to inhibit topoisomerase II, a DNA replication enzyme, in *Giardia lamblia *[9,15]. Also, DB75 inhibits cellular respiration in the mitochondria of *Sacchharomyces cerevisiae *and trypanosomes [13] Finally, DB75 localizes in acidocalcisomes of trypanosomes where it may interrupt calcium homeostasis [14]. Plasmodium also possess acidocalcisomes that regulate calcium homeostasis in a manner similar to other protozoan [16].

The objective of this investigation was to elucidate the mechanism of action for DB75 in *P. falciparum*.

## Methods

### Parasite cultivation

3D7 *P. falciparum *strain was maintained with 2% (v/v) O^+ ^human erythrocytes (Research Blood Components, Brighton, MA) in 1640 RPMI supplemented with 25 mM HEPES (Sigma), 2 mM L-glutamine (Gibco), 0.45% (w/v) glucose (Sigma), 0.05 ng/ml gentamycin (Sigma), 0.1 mM hypoxanthine and 10% (v/v) O^+ ^human serum (Research Blood Components). Cultures were maintained by the Trager and Jensen method in candle jars at 37°C [17].

### [^3^H]-Hypoxanthine incorporation assay

[^3^H]-hypoxanthine incorporation into parasite DNA was measured in the presence and absence of DB75 to assess parasite drug susceptibility in vitro [18]. Parasite cultures were diluted to 0.7% parasitaemia in complete medium (without supplemental hypoxanthine). Parasites were sub-cultured in 96-well flat bottom microtitre plates with serial dilutions of DB75 at final concentrations ranging from 1 μM to 3.9 nM, in duplicate or triplicate.

To measure growth in 42 hours, the 96-well plates were incubated for 24 hours prior to addition of 0.5 μCi of [^3^H]-hypoxanthine diluted in hypoxanthine-free complete media (25 μL/well). The cells were incubated for an additional 18 hours and harvested using an Inotech cell harvester and glass fiber filter paper (Inotech Systems International, INC.). Radioactivity of samples was determined in a liquid scintillation counter (Beckman-Coulter) [18]. Background absorption of [^3^H]-hypoxanthine by erythrocytes was controlled for using uninfected erythrocytes, and maximum parasite growth was controlled for using non-treated parasites.

To measure growth in 36 hours, [^3^H]-hypoxanthine was added for the final 18-hour incubation. To measure growth in 96 hours, 25 μL of medium was removed at 48 hours and replaced with fresh medium containing [^3^H]-hypoxanthine and DB75 for the final 48-hour incubation.

DB75 concentration required to inhibit 50% of parasite growth (IC50) was calculated using sigmoidal dose response nonlinear regression equation in GraphPad Prism^® ^(version 4.0). The mean IC50 ± SEM for 42-hour growth was calculated from 12 experiments. For 36- and 96-hour experiments, the IC50 ± SEM was calculated from two experiments.

### Confocal microscopy

Asynchronous parasite-infected red blood cells were stained with fluorescent dyes for subcellular localization studies. Cells were treated with MitoTracker Red CMXRos (Invitrogen), which is a mitochondrion-specific dye or LysoTracker^® ^Red DND-99 (Invitrogen), an acidophilic dye that localizes to the parasite food vacuole [19,20]. Cells were also treated with Draq5™ (Axxora, LLC), a nuclear DNA dye in addition to the inherently fluorescent compound, DB75 [21].

To determine if DB75 co-localizes with MitoTracker Red, red blood cells infected with viable asynchronous parasites suspended in medium were incubated with 25 nM MitoTracker Red for 30 minutes at 37°C in a candle jar. The cells were centrifuged and resuspended in pre-warmed complete medium to remove the mitochondrial dye from the extracellular medium. 1 μM DB75 was added and cells were transported to the Michael Hooker Microscopy facility (15 minutes) where 1 μM of the DNA dye was added immediately prior to slide preparation. To determine if DB75 co-localizes with LysoTracker Red, infected red blood cells were prepared as described above except cells were incubated with 50 nM of LysoTracker.

To assess DB75 co-localization with Draq5™, 1 μM DB75 was added to infected red blood cells suspended in medium prior to transport to the microscopy location. 1 μM of the nuclear dye was added immediately prior to slide preparation.

To determine subcellular localization with short-term drug exposure, infected red blood cells (> 5% parasitaemia) were incubated with 1 μM DB75 for less than four hours prior to microscopy. For long-term drug exposure, infected red blood cells (3% parasitaemia) were exposed to 100 nM DB75 for 24, 48 or 72 hours. Dyes for co-localization with DB75 were added after the long exposure periods to determine the subcellular localization of the drug. To determine if DB75 remains in the parasite after extracellular drug pressure is relieved, parasites were exposed to DB75 for 24 hours and then washed by centrifugation and resuspended in drug-free pre-warmed medium. The parasites were incubated for an additional 48 hours prior to microscopy.

For confocal microscopy, live cells were examined under a cover slip using a Leica 2SP Laser Scanning Confocal Microscope. The 561 nm Solid State laser was used to capture MitoTracker (absorbance/emission: 579 nm/599 nm) or LysoTracker (absorbance/emission: 577 nm/590 nm); a UV laser (364 nm) for DB75 (absorbance/emission 365 nm/465 nm) and Red HeNe laser to capture Draq5™ (absorbance/emission: 647 nm/670 nm). A spectral scan was used to identify changes in the emission spectrum of DB75. Adobe Photoshop (v.7.0) was used to create overlaid confocal images and to adjust dye contrast for publication.

### Parasite maturation assay

To determine the effect of DB75 on parasite development, morphology was assessed after prolonged drug exposure. Parasite cultures were synchronized every 48 hours for three consecutive life cycles prior to a 24–48 hour recovery period [22]. Synchronized ring or trophozoite parasites (0.3% parasitaemia) were exposed to DB75 for 96 hours. Medium for each sample was exchanged at 48 hours with fresh medium and DB75. Non-treated synchronized cultures were used as a control. Thin blood smears were prepared and stained with Giemsa at 0, 12, 24, 36, 48, 60, 72, 84 and 96 hours post-exposure and examined with light microscopy to assess parasitaemia and parasite morphology. Parasitaemia was determined by calculating the number of parasite-infected red blood cells per at least 1000 total red blood cells. Parasite morphology was examined and the number of rings, trophozoites and schizonts was assessed for at least 100 parasites. Parasites with the "ring" structure and a single nucleus were classified as ring stage parasites. Parasites containing a single nucleus and haemozoin were classified as trophozoites. Multi-nucleated parasites with ample haemozoin were classified as schizonts. Results are representative of three separate experiments.

### Gene expression

Red blood cells infected with sorbitol-synchronized rings were exposed to 100 nM or 500 nM DB75 for 48 hours. Non-treated cells were used as a control. At 0, 6, 12, 18, 24, 36 and 48 hours, RNA and DNA were immediately extracted from the harvested cells using the Allprep kit (Qiagen). Also, blood smears were prepared for assessment of morphology and parasitaemia at each time point. RNA concentrations were measured using a NanoDrop 1000 (NanoDrop Technologies). 50 ng of total RNA was reverse transcribed using SuperScript^® ^II (Invitrogen) with 2.2 μM Pd(N)_6 _Random Hexamer (GE Healthcare) and 2.2 μM Oligo(dT)20 primers (Invitrogen) according to the manufacturer's protocols. All cDNA samples were diluted by a factor of 50 in sterile, RNase free water, and 5 μL was subsequently used for Real Time PCR to measure expression in six developmentally-regulated genes. Primers were designed using Primer Express v3.0 (Applied Biosystems) (Additional file 1).

Real Time PCR was performed using an Applied Biosystems 7300 Real-Time PCR System (Applied Biosystems) with Power SYBR^® ^Green PCR Master Mix (Applied Biosystems). cDNA was amplified with 40 cycles of: 95° for 15", 55° for 30", 72° for 30". A dissociation curve for amplicon melting temperature analysis was performed immediately following amplification to monitor nonspecific amplification (contamination or primer dimer amplification).

The expression of each gene relative to a constitutively expressed gene was analysed for each sample [23]. Expression of six developmentally expressed genes were assessed: *trophozoite antigen R45-like *(ring stage), *lactate dehydrogenase *(late ring stage), *DNA primase *(trophozoite stage), *isocitrate dehydrogenase *(trophozoite stage), *merozoite surface protein-1 *(schizont stage), *merozoite surface protein-7 *(schizont stage) (Additional file 1) [24]. In replicates on the same reaction plate, amplification of cDNA for a developmentally-regulated gene was compared to that of the constitutively expressed, 18s rRNA. The amplification efficiency for each primer pair was calculated based on the slope of the line representing cycle threshold (CT) values versus 3D7 genomic DNA concentrations from five separate experiments as previously described [23]. To determine the relative fold change in expression over time for each developmentally-regulated gene, the following equation was used [23]:



Where E is primer efficiency, CT is the cycle threshold, *i *is the developmentally-regulated gene of interest, *t *is the time of drug exposure, *t = 0 *is the start of the experiment and *ref *is the 18s rRNA reference gene [23]. This method accounts for variations in cDNA concentrations (loading control) and differences in primer efficiency. Data were graphed using GraphPad Prism^® ^(v.4.03). Results are representative of two separate experiments.

## Results and discussion

### DB75 inhibits *P. falciparum *growth

**Figure 1:**
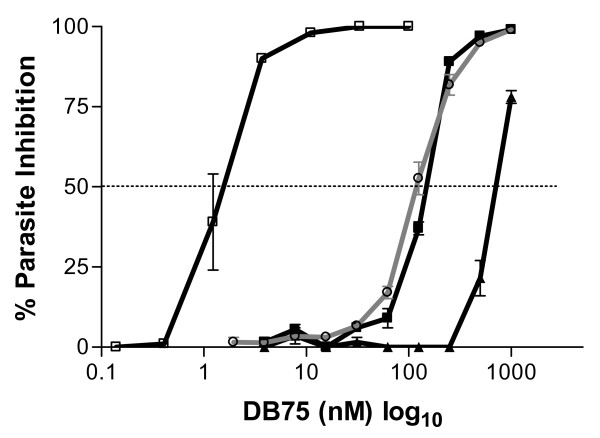
**DB75 inhibition of *P. falciparum***. The percent inhibition of parasite growth was measured with tritiated hypoxanthine for asynchronous parasites exposed to drug for 42 hours (grey line, open circle); synchronized ring-stage parasites exposed to drug for 36 hours (black line, closed square); synchronized rings-stage parasites exposed to drug for 96 hours (black line, open square); and synchronized trophozoite-stage parasites exposed to drug for 36 hours (black line, closed triangle).

To determine the anti-malarial activity of DB75 in vitro, tritiated hypoxanthine incorporation was measured. DB75 had an IC50 of 124 ± 10 nM (mean ± SD) with asynchronous parasites exposed for 42 hours (Figure 1). When synchronized ring and trophozoite cultures were exposed to DB75 for 36 hours, the IC50 for rings was 143 ± 8 nM compared to 711 ± 32 nM for exposed trophozoites (mean ± SEM), suggesting that rings are particularly sensitive to DB75 (Figure 1). The IC50 was reduced to 1.5 ± 0.2 nM for synchronized rings exposed for 96 hours, suggesting longer incubations increase DB75 inhibitory activity (Figure 1).

### Fluorescent localization of DB75 in *P. falciparum*

**Figure 2:**
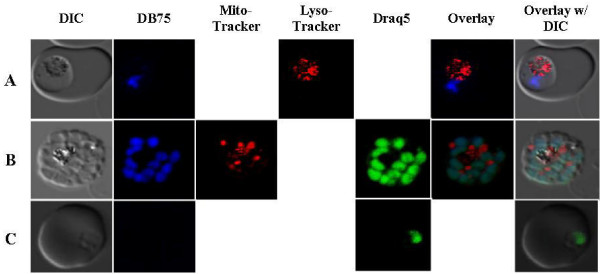
**DB75 subcellular distribution in different *P. falciparum *life stages upon immediate cell entry (< 4 hours)**. (A) 1 μM DB75 (blue) and LysoTracker Red show no co-localization of the food vacuole of a trophozoite. (B) In a schizont, 1 μM DB75 co-localizes with green nuclear Draq5™ stain but not MitoTracker Red. (C) 1 μM DB75 fluorescence is not present in ring stage parasites. All images are representative.

The intrinsic fluorescent properties of DB75 were used to determine its potential subcellular localization in *P. falciparum *using confocal microscopy. To identify possible sub-cellular targets, infected red blood cells were incubated with DB75 and different organelle-specific dyes [19-21]. In parasites exposed to 100 nM or up to 1 μM drug for less than four hours, DB75 co-localized only with Draq5™, the nuclear DNA stain. Nuclear co-localization was evident in all parasite life stages except the ring stage with short-term exposure (Figure 2), suggesting that the drug initially localizes in the nucleus and may require a stage-specific uptake mechanism that prevents accumulation in ring stage parasites. DB75 had a distinct and separate staining pattern from the red-fluorescing LysoTracker or MitoTracker, suggesting that DB75 does not co-localize with these dyes in the food vacuole or mitochondrion immediately after drug exposure. 3-D compilations of z-series images confirmed DB75 co-localized only with Draq5™ and not LysoTracker or MitoTracker (Additional files 2 and 3).

**Figure 3:**
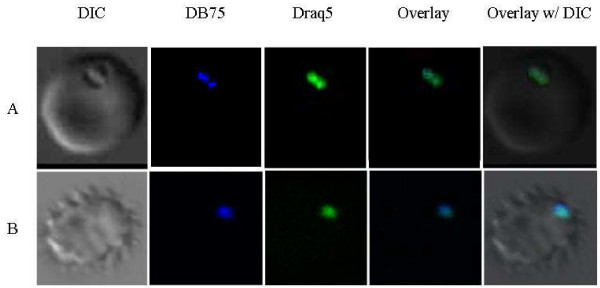
**DB75 subcellular distribution following long-term drug exposure**. (A) Trophozoite-infected erythrocytes after exposure to 100 nM DB75 for 24 hours. Note co-localization of DB75 and nuclear DNA stain, Draq5™. (B) Drug was removed by washing at 24 hours and infected erythrocytes incubated for an additional 48 hours in drug-free medium. Note persistent co-localization of DB75 and Draq5™ in nucleus of pycnotic infected erythrocyte.

To determine if DB75 localizes to other subcellular compartments over time, asynchronous parasites were exposed to a therapeutic concentration (100 nM) of DB75 for 24, 48 or 72 hours. Despite the longer incubation, DB75 co-localized only with Draq5™ following 24, 48 or 72 hours, suggesting the drug targets the nucleus of the parasites with extended exposures (Figure 3). DB75 co-localization with Draq5™ was noted in all life stages after 24 hours of exposure, including rings. Although DB75 was not evident in ring-stage parasites with short-term drug exposure, the presence of DB75 in rings after extended drug exposure suggests DB75 may not be transported into the cell during the early part of the life stage. However, with extended exposure DB75 localization in the nucleus of schizonts likely persists as the host cell ruptures and merozoites invade uninfected erythrocytes and mature to the ring stage.

**Figure 4:**
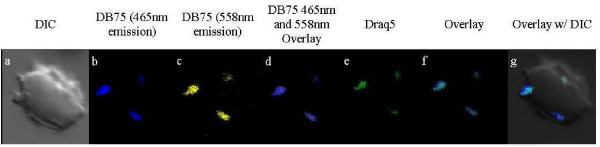
**DB75 distribution in an erythrocyte infected with multiple parasites following 72 hours of exposure**. DB75 emits at both 465 nm (b) and 558 nm (c) with extended exposure. The fluorescence emitted at these wavelengths overlay with Draq5™ (f and g). Shown are red cells infected with crisis forms.

After 48 or 72 hours of continuous exposure of infected red blood cells to 100 nM DB75, the drug's emission spectrum undergoes a shift from 465 nm (blue) to 558 nm (yellow), although still localized to the nucleus (Figure 4). This spectral shift may be the result of high concentrations of the compound, as is seen with the DNA-binding dye, Hoechst [25]. A similar shift from blue to yellow fluorescence is also observed by epifluorescence in trypanosomes exposed to 7.5 μM DB75 [14].

To determine if and where DB75 persists in the cell after drug pressure was removed, live cells were assessed with co-localization dyes 48 hours after drug was removed. When *P. falciparum *cells were washed after 24 hours of continuous exposure to 100 nM DB75, the blue DB75 fluorescence was still observed in the nucleus of cells at 48 and 72 hours post-exposure by colocalization with Draq5™. However, only the blue fluorescence (465 nm emission) was detected (Figure 3). This suggests that the drug remains in the nucleus, perhaps bound to DNA, but does not accumulate to high levels that may result in the fluorescence shift from blue to yellow.

All DB75-treated parasites appeared to be unhealthy or dead following 72 hours of continuous exposure to 100 nM DB75 (Figure 4). Many parasites were outside of the host red blood cells, and all exhibited condensed nuclei.

### Effects of DB75 on parasite maturation

**Figure 5:**
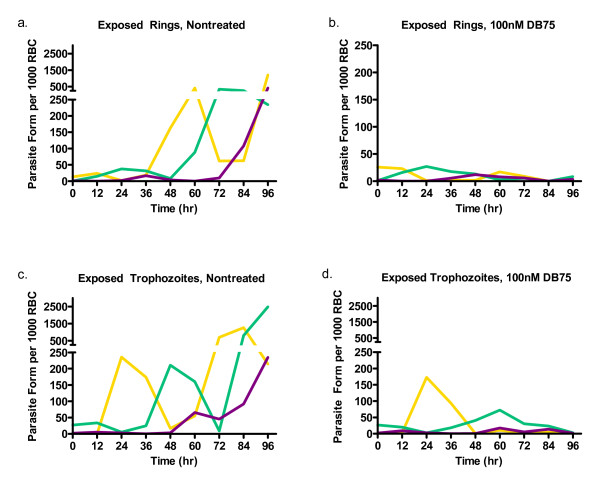
**Parasite morphology with 96 hour continuous DB75 exposure**. (a and b) Synchronized rings or (c and d) trophozoites (a and c) without treatment or (b and d) with exposure to 100 nM DB75 (b and d) for 96 hours. The number of ring (yellow), trophozoite (green) and schizonts (purple) are shown per 1000 red blood cell over time. Note: the x-axis of (b) is on a different scale.

The development of different parasites stages over 96 hours, under normal conditions, can be seen in Figure 5a. Untreated cultures consisting of synchronized rings progressed through two lifecycles, to cultures which were predominantly trophozoites (peaking at 24 hours) schizonts (36 hours), then rings (60 hours), trophozoites (72 hours) and schizonts (≥ 96 hours) again. However, when synchronized rings were exposed to DB75, parasites matured into the first wave of trophozoites and schizonts normally, but not into the second wave of rings or subsequent stages (Figure 5b).

The situation was different for experiments initiated with parasites synchronized at the trophozoite stage. Untreated cultures of trophozoites also progressed through two life cycles as expected (Figure 5c). When cultures were exposed to DB75, there was no effect on the timing or magnitude of the first ring population. However, there was a significant delay in the appearance of the second trophozoite population, which peaked at 60 hours instead of 48 hours as in untreated parasites. Furthermore, few schizonts or rings appeared after this peak in the treated cultures.

### DB75 effect on gene transcription

The effect of drug on transcription was measured for six developmentally regulated genes (Additional file 1) [24]. With synchronized ring-stage parasites, the transcription profiles were assessed for alterations in genes expressed during specific life stages relative to a constitutively-expressed gene.

**Figure 6:**
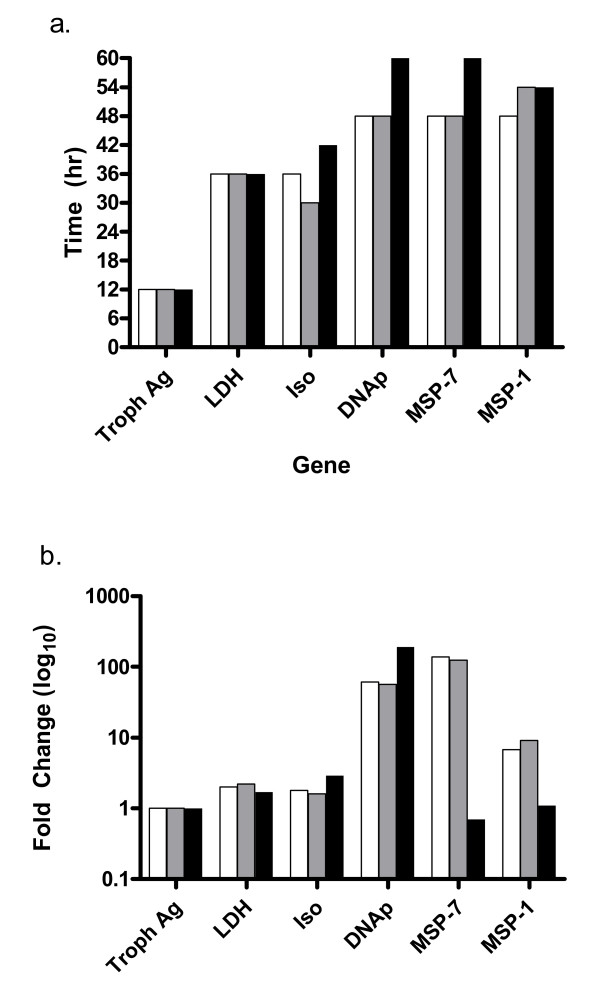
**Effect of DB75 on peak expression time and intensity**. Expression profiles for six developmentally expressed genes were compared in synchronized rings with exposure to 100 nM (grey) or 500 nM (black) DB75 or left untreated (white). (a) Time of invasion was approximated based on assessment of morphology, and peak expression post-invasion was estimated for all genes. (b) Expression intensity at time of peak expression was measured as the fold change in expression relative to the constitutively expressed 18s rRNA for exposed rings. Genes evaluated include: *trophozoite antigen r45-like *(Troph Ag); *lactate dehydrogenase *(ldh), *isocitrate dehydrogenase *(Iso);*DNA primase *(DNAp), *Merozoite surface protein-1 *(MSP-1); *Merozoite surface protein-7*(MSP-7).

For genes normally expressed during the early life stages, *trophozoite antigen R45-like *and *lactate dehydrogenase*, peak expression occurred in the first 12 hours following invasion for untreated rings (Figure 6a). A similar pattern was observed for cells exposed to 100 nM or 500 nM DB75 (peak expression, 12 hours). The fold change in expression (peak intensity) relative to 18s rRNA for these early transcribed genes varied little with DB75 exposure. The peak intensity in non-treated cells was 2.0 for *lactate dehydrogenase *and 2.2 or 1.7 with addition of 100 nM or 500 nM DB75, respectively (Figure 6b). Similarly, peak intensity for *trophozoite antigen R45-like *was 1.0 for both non-treated and treated cells. The DB75 treatment had little or no effect on the timing or intensity of expression of these early genes.

The effects of DB75 were also measured for two trophozoite-specific transcripts: *isocitrate dehydrogenase *or *DNA primase*. Peak expression for these genes in untreated rings occurred later than expected at 36 and 48 hours, respectively (Figure 6a). Unlike the ring-stage genes, peak expression for the trophozoite-specific transcripts was altered with addition of drug. With 100 nM DB75, the expression of *isocitrate dehydrogenase *occurred in exposed rings at 30 hours post-invasion, which is six hours earlier than non-treated cells (Figure 6a). The highest concentration of DB75 had the opposite effect. Peak expression was delayed six hours to 42 hours post-invasion. 500 nM DB75 also delayed expression of *DNA primase *by 12 hours relative to non-treated cells (60 hours). The delays observed with the highest concentration of DB75 were consistent with the delay in maturation.

The intensity of peak expression for both *isocitrate dehydrogenase *and *DNA primase *with 500 nM DB75 was increased (Figure 6b). This is especially evident for DNA primase where there was a 61-fold increase in expression in non-treated cells but 192-fold increase with addition of 500 nM DB75. The lower concentration of DB75 did not have a pronounced effect on peak intensity for either *isocitrate dehydrogenase *or *DNA primase *(57-fold) compared to non-treated cells (Figure 6b). Thus, the expression of the trophozoite-stage genes are both delayed and increased by 500 nM drug.

The effect of drug was also measured on genes expressed during the schizont stage, *merozoite surface protein-1 *and *merozoite surface protein-7*. For non-treated ring stage cells, peak expression occurred at 48 hours post invasion, when parasites were at the schizont stage. There was no change in *merozoite surface protein-7 *peak expression time with 100 nM DB75 (48 hours) (Figure 6a). However, the same concentration of DB75 delayed peak expression of *merozoite surface protein-1*. Additionally, 500 nM DB75 delayed the expression of both schizont-stage genes by 6 or 12 hours. Also, 500 nM DB75 drastically reduced peak intensity of these late-stage genes. *Merozoite surface protein-1 *expression was reduced from a 139-fold increase to 0.7 (Figure 6c). *Merozoite surface protein-7 *peak expression intensity was reduced from a 6.8 fold increase to 1.1. Again, this is consistent with the delayed maturation effect observed above.

Taken together, DB75 does not appear to enhance or suppress selected genes whose expression is associated with specific lifecycle stages. However, delays in peak expression for mid- and late-stage expressed genes were observed, and are consistent with the delays observed by microscopy.

DB75 is representative of a class of newly synthesized diamidine compounds with anti-malarial activity. Confocal microscopy demonstrates that DB75 co-localizes with a nuclear DNA dye in the nucleus after both short (≤ 4 hours) and long exposures (24–72 hours) but not with markers for other organelles, suggesting that DB75 may target the nucleus. The absence of co-localized staining with dyes for the mitochondrion or food vacuole argues against these organelles as targets for DB75; however, due to the complicated nature of fluorescence in the cell, these organelles cannot be completely excluded.

By microscopy, DB75-treated cultures tended to stall in the trophozoite stage. By quantifying transcription of six stage specific genes, it is evident that DB75 causes delays in expression but not inhibition of stage-specific transcripts. Thus, both morphological and molecular evidence suggests that DB75 may stall parasite maturation.

Previous reports suggest that the nucleus may be a target for DB75. Epifluorescence and confocal microscopy studies show DB75 localizes to the nucleus and mitochondria of tumor cells [14,26]. Additional studies show DB75 binds to the minor groove of DNA at 5'-AATT-3' [27]. Since the nuclear genome of *P. falciparum *is 80% AT rich, the parasite genome may contain a high number of these sites [28]. Thus, DB75 selectively accumulates in the nucleus of both mammalian cells and *P. falciparum*.

The data suggest that DB75 has a different mechanism of action in malaria than in trypanosomes. In trypanosomes, epifluorescence experiments show that DB75 accumulates in the mitochondria, acidocalcisomes, nucleus, and kinetoplast [15,29]. Based on these observations, the accumulation of DB75 in the nucleus, mitochondrion and/or acidocalcisomes of Plasmodium was anticipated. However, the results suggest that subcellular localization of DB75 is limited to the nucleus of the *P. falciparum *parasite. While other subcellular sites cannot be eliminated from consideration based solely on DB75 fluorescence, the data presented are consistent with the observation that DB75 does not affect malarial mitochondria (Akhil Vaidya, personal communication). Further molecular studies may eliminate non-nuclear targets.

The results are not consistent with the mechanism of action for diamidines proposed by Stead et al., who suggest that diamidine compounds act by targeting the haemozoin synthesis pathway in the food vacuole [12]. In solution, 3 μM pentamidine binds haeme and prevents haemozoin formation as determined by spectroscopy. With confocal microscopy, DB75 does not accumulate in the food vacuole with short term or long-term drug exposure. One possible explanation for this lack of appearance of drug in the food vacuole is fluorescent quenching at low pH. However, DB75 fluorescence is not quenched by low pH (J. Ed Hall, personal communication). Further studies using electron micrographs or isotope-labelled DB75 are needed to definitively rule out the food vacuole as a target.

The effects of DB75 on parasite maturation might, in part, be mediated by inhibition of genes transcribed early in the life cycle when DB75 exposure appears to be critical. A similar stage-specific inhibition was observed with the anti-malarial compound, hexadecyltrimethylammonium bromide, which inhibits late-stage expression of *Plasmodium falciparum choline kinase (pfck) *[30].

Since only six stage-specific genes were tested, it is possible that DB75 may exert some specific effect on genes not tested. A full microarray analysis may identify such specificity if it exists in *P. falciparum*.

The increased *DNA primase *expression was unexpected. DNA primase is required for DNA synthesis [31] and further studies are needed to determine whether this is a general response to cell death or is related to the drug mechanism of action. DNA primase translational regulation should also be investigated since DB75 binds to single stranded RNA and could affect post-transcriptional modification or translation [32].

While these data suggests that ring-infected parasites may be more sensitive to DB75 than trophozoites, a recent study by Hofer *et al *[33] suggests that trophozoites may be more sensitive. However, this study used lower concentrations and longer exposures than the Hofer *et al *study, so a direct comparison cannot be made.

This study had a few limitations. First, synchronization of parasites is imperfect. Parasites are synchronized at the ring stage and examination of morphology shows > 90% of the parasite population is at the desired state at the start of experiments. The remaining unsynchronized parasites may slightly skew gene expression and morphology results. Second, classification by morphology of parasite life stage is only approximate and imperfect. To minimize this, the samples were blinded to prevent bias and all morphology assessments were determined by one individual. Third, the results presented here show that DB75 localizes in the nucleus, but the authors cannot definitively say that this is the single target in *P. falciparum*. DB75 may exhibit unknown fluorescent properties, such as quenching, in the presence of biological substrates. Therefore, it cannot be concluded that the compound is not present when the fluorescence may, in fact, be quenched. Finally, the complicated nature of capturing images of live parasite-infected erythrocytes with confocal microscopy limited the number of cells we were able to examine.

## Conclusion

Taken together, the results presented here suggest that DB75 may target the nucleus and nucleic acid synthesis. DB75 localizes in the nucleus and slows the maturation of parasites. This is evident by the stall in development at the trophozoite stage. In addition, DB75 must be present in the early life stages to interrupt nuclear activity in the cell. Inhibition of developmental gene transcription is not likely to be the mechanism of action because alterations with gene expression profiles occur only with the highest concentration of DB75 and appear to be a result of the stall in maturation. However, this study only examined DB75's effect on the transcription of six select genes; future studies should investigate the full extent of the drug's effect on developmental gene expression using microarrays. In addition, the effect of DB75 on DNA synthesis, nuclear enzyme activity and mRNA translation should be examined to further elucidate the mechanism of action. This is the first time the mechanism for anti-malarial action of DB75 has been evaluated. Results obtained in this study are potentially relevant to the clinical application of DB289. Also, these results may translate to other dicationic diamidine compounds in development for anti-malarial use.

## Competing interests

The authors declare that they have no competing interests.

## Authors' contributions

AP contributed to the study design, data collection and analysis, interpretation of results and preparation of the manuscript.

RT contributed to the preparation of the manuscript.

SM contributed to the study design, interpretation of results and preparation of the manuscript.

## Supplementary Material

Additional file 1 **Developmentally expressed genes assessed for changes with DB75 exposure**. Primers and stage specificity of 6 gene transcripts. Click here for file

Additional file 2 **3-D compilation of z-series images show DB75 localization is distinct from mitochondria**. 3-D co-localization of DB75 with Draq5™ (aqua color) is distinct from red mitotracker (mitochondria) in *P. falciparum *schizont. Video may be viewed with Windows Media Player. Click here for file

Additional file 3 **3-D compilation of z-series images show DB75 localization is distinct from food vacuole**. 3-D localization of DB75 (blue) is distinct from red LysoTracker (food vacuole) in *P. falciparum *trophozoite. Video may be viewed with Windows Media Player. Click here for file
