# Supplementary material for: The diamidine DB75 targets the nucleus of Plasmodium falciparum
Source: Malar J. 2009 May 14;8:104. doi: 10.1186/1475-2875-8-104 (PMC2689252; doi:10.1186/1475-2875-8-104)
Supplement: Additional file 1 — Developmentally expressed genes assessed for changes with DB75 exposure. Primers and stage specificity of 6 gene transcripts. [file 1475-2875-8-104-S1.doc]

| Reverse Primer (5’-3’) | CATTTAAGTCTGTCTTCATTCTACTTCT | TCTCTATTCCATTCTTTGTCACTCTTC | TTTTCTCCCTTTTGATAAGCTCTA | GTTGTCTTCCCCAATGTTTTAA | TGAAATGTCGATTCTCCTCCTT | TTGTTACCGTTGTTGTGTGTCCTA | GGGTAGGAGTACTCATAATATGTAGA |
| --- | --- | --- | --- | --- | --- | --- | --- |
| Forward Primer (5’-3’) | ACGAGCTGACCCACCAAA | ACGATTTGGCTGGAGCAGAT | CCAGACGGTGTTACGTGTGT | AGATTGTCTCCTCAAAACCTTTATAG | CAAAGTGAAACAGATACTCAATCTAAAA | AGCAAGCGAAACAACTGAAGA | GGCAACTGTTATATGTAAACAAGCA |
| Stage  Expressed | Ring | Late Ring/  Early Trophozoite | Trophozoite | Trophozoite | Schizont | Schizont | Constitutive |
| ^*^Maximum  Expression (hr) | 16 | 21 | 29 | 30 | 37 | 42 |  |
| Gene (PlasmoDB ID) | *Trophozoite Antigen r45-like protein* [GenBank:PFD1175w] | *L-lactate dehydrogenase*  [GenBank:PF13_0141] | *Isocitrate dehydrogenase*  [GenBank:PF13_0242] | *DNA primase* [GenBank:PFI0530c] | *Merozoite surface protein 7*  [GenBank:PF13_0197] | *Merozoite surface protein 1*  [GenBank:PFI1475w] | *Ribosomal protein S18*  [GenBank:PF11_0272] |

*Time post-invasion for maximum transcript levels detected by microarray (ref 24).
